# ALCAM Mediates DC Migration Through Afferent Lymphatics and Promotes Allospecific Immune Reactions

**DOI:** 10.3389/fimmu.2019.00759

**Published:** 2019-04-12

**Authors:** Ann-Helen Willrodt, Ann-Charlott Salabarria, Philipp Schineis, Desislava Ignatova, Morgan Campbell Hunter, Martina Vranova, Alexandra M. Golding-Ochsenbein, Elena Sigmund, Annatina Romagna, Verena Strassberger, Marina Fabbi, Silvano Ferrini, Claus Cursiefen, Dario Neri, Emmanuella Guenova, Felix Bock, Cornelia Halin

**Affiliations:** ^1^Institute of Pharmaceutical Sciences, ETH Zürich, Zurich, Switzerland; ^2^Department of Ophthalmology, University of Cologne, Cologne, Germany; ^3^Department of Dermatology, University Hospital of Zürich, University of Zurich, Zurich, Switzerland; ^4^IRCCS Ospedale Policlinico San Martino, Genoa, Italy; ^5^Center Molecular Medicine Cologne (CMMC), University of Cologne, Cologne, Germany

**Keywords:** ALCAM, dendritic Cell (DC), lymphatic vessel, lymphangiogenesis, DC migration, cornea, allograft rejection, blocking antibody

## Abstract

Activated leukocyte cell adhesion molecule (ALCAM, CD166) is a cell adhesion molecule of the immunoglobulin superfamily and has been implicated in diverse pathophysiological processes including T cell activation, leukocyte trafficking, and (lymph)angiogenesis. However, exploring the therapeutic potential of ALCAM blockade in immune-mediated inflammatory disorders has been difficult due to the lack of antibodies with blocking activity toward murine ALCAM. In this study, we identified and characterized a monoclonal antibody with high affinity and specificity for murine ALCAM. This antibody reduced *in vitro* T cell activation induced by antigen-presenting dendritic cells (DCs) as well as (trans)migration of murine DCs across lymphatic endothelial monolayers. Moreover, it reduced emigration of DCs from *in vitro*-cultured human skin biopsies. Similarly, antibody-based blockade of ALCAM reduced (lymph)angiogenic processes *in vitro* and decreased developmental lymphangiogenesis *in vivo* to levels observed in ALCAM-deficient mice. Since corneal allograft rejection is an important medical condition that also involves (lymph)angiogenesis, DC migration and T cell activation, we investigated the therapeutic potential of ALCAM blockade in murine corneal disease. Blocking ALCAM lead to DC retention in corneas and effectively prevented corneal allograft rejection. Considering that we also detected ALCAM expression in human corneal DCs and lymphatics, our findings identify ALCAM as a potential novel therapeutic target in human corneal allograft rejection.

## Introduction

Cell adhesion molecules are central players in vascular biology and immunology: they regulate vascular stability and permeability and also control leukocyte activation and recruitment into tissues. ALCAM (also known as CD166) is a 100–110 kDa adhesion molecule of the immunoglobulin superfamily that is expressed by various cell types including leukocytes and endothelial cells (ECs) (1, 2). ALCAM was shown to bind to the T cell-expressed scavenger receptor CD6 (3), to endothelial L1CAM (4), and to galectin-8 (5). In addition, low-affinity homophilic ALCAM-ALCAM interactions have been described (1). ALCAM reportedly supports *in vitro* transmigration of activated T cells (6) and monocytes (7, 8) across EC monolayers. Moreover, ALCAM on DCs interacting with T cell-expressed CD6 was shown to provide T cell co-stimulation *in vitro* (9). In line with the latter findings, two recent studies reported that ALCAM-deficient (ALCAM^−/−^) mice are partially protected from T cell-mediated inflammation in murine models of asthma (10) and food allergy (11). In ECs ALCAM was shown to mediate *in vitro* migration, tube formation and barrier function of blood vascular and lymphatic ECs (LECs) (2, 12, 13). Moreover, our group recently demonstrated a role for ALCAM in the formation of both vascular networks *in vivo* (12, 14) and in tumor angiogenesis (14), whilst another study reported that ALCAM regulates the integrity of the blood brain barrier (13).

Given the involvement of ALCAM in leukocyte trafficking, (lymph)angiogenesis, and the induction of T cell-mediated immune responses, therapeutic blockade of ALCAM with monoclonal antibodies could represent a promising approach for treating immune-mediated inflammatory disorders. A pathologic condition that involves all of the above-mentioned processes is allograft rejection. Corneal allografts are among the most commonly transplanted tissues and are typically well tolerated (15, 16). Under normal conditions the cornea is avascular due to the expression of potent anti-(lymph)angiogenic factors (15, 16). However, the presence of inflammation-induced neovascularization in the recipient's cornea prior to transplantation is nowadays well recognized to significantly increase the risk of allograft rejection (17–19). Under such pre-vascularized conditions, blood vessels mediate leukocyte recruitment, and lymphatic vessels provide the exit routes for alloantigen-presenting dendritic cells (DCs), which migrate to draining lymph nodes to induce T-cell mediated allograft rejection (15, 16). Particularly the presence of inflammation-induced lymphatic vessels in the recipient cornea was shown to significantly increase the risk of corneal allograft rejection (17–19).

In this study we reformatted a previously described single-chain variable fragment (scFv) antibody with blocking activity toward human ALCAM (20) into a bivalent Fc fusion protein (I/F8-Fc) and validated its ability to bind and block murine ALCAM *in vitro* and *in vivo*. Using a murine model of corneal transplantation we identified ALCAM as a novel mediator of DC migration and as a promising target for preventing allograft rejection.

## Results

### Engineering and Characterization of I/F8-Fc

**Figure 1:**
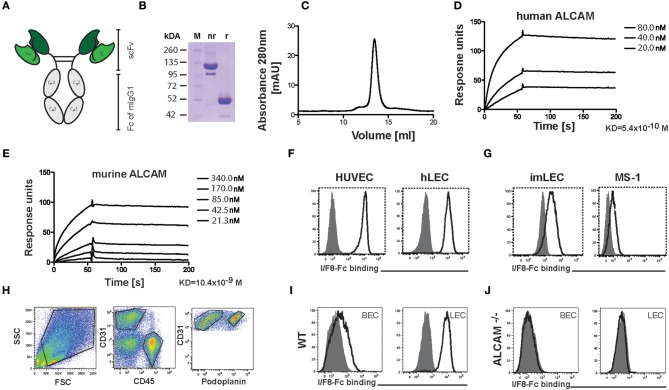
Production and characterization of the ALCAM-blocking antibody I/F8-Fc. **(A)** Schematic representation of the homodimeric I/F8-Fc antibody, consisting of scFv IF/8 fused to the Fc portion of murine IgG_1_. **(B)** SDS-PAGE of I/F8-Fc under non-reducing (nr) and reducing (r) conditions. Expected molecular weight: 51 kDa under reducing conditions. **(C)** In FPLC I/F8-Fc elutes as a homodimer. **(D,E)** Surface plasmon resonance profiles of purified I/F8-Fc on micro sensor chips coated with **(D)** human and **(E)** murine ALCAM. **(F,G)** FACS analysis reveals I/F8-Fc binding to **(F)** human and **(G)** murine endothelial cell lines. **(H–J)** Analysis of I/F8-Fc specificity by FACS analysis performed on single cell suspensions generated from murine lungs of WT and ALCAM^−/−^ mice. **(H)** FACS gating strategy for differentiating BECs from LECs. Single-cell suspensions from enzymatically digested lung tissue were stained for CD45, CD31, and podoplanin. BECs and LECs were identified, by gating on CD45^−^CD31^+^podoplanin^−^ (BECs) or CD45^−^CD31^+^podoplanin^+^ (LECs) cells. **(I,J)** I/F8-Fc stains BECs (CD45^−^CD31^+^podoplanin^−^) and LECs (CD45^−^CD31^+^podoplanin^+^) in lung single-cell suspension derived from **(I)** WT mice but not from **(J)** ALCAM^−/−^ mice. Black lined histogram: I/F8-Fc, shaded histogram: KSF-Fc control. Representative data from one out of four similar experiments are shown in **(F–J)**.

To date no monoclonal antibody with biochemically documented blocking activity toward murine ALCAM has been described. Treatment with polyclonal goat anti-mouse ALCAM antibody was recently shown to reduce allergic symptoms in a murine asthma model (10), but polyclonal antibodies from other species are not suited for long-term therapies due to immunogenicity. In addition, treatment with a monoclonal mouse IgG_1_ raised against human ALCAM was previously reported to reduce the severity of murine experimental autoimmune encephalitis (6), but in our hands this antibody neither blocked nor bound murine ALCAM (Figure S1). Given the 93.5% amino acid identity between human and murine ALCAM, we investigated whether I/F8, a previously described antibody in single-chain variable fragment (scFv) format with blocking activity toward human ALCAM (20), would block murine ALCAM. For this, we reengineered scFv I/F8 into a bivalent Fc fusion protein (I/F8-Fc), in order to increase its functional avidity and half-life (Figures 1A–C and Figures S2A,B). In parallel, the isotype control antibody KSF-Fc with specificity for hen egg lysozyme (21) was generated (Figures S2A,C). In surface plasmon resonance measurements I/F8-Fc bound to human and murine ALCAM with dissociation constants in the nanomolar range (Figures 1D,E). FACS analysis confirmed the ability of I/F8-Fc to bind ALCAM on primary human and murine ECs (Figures 1F,G). To investigate the specificity of I/F8-Fc binding, we generated single-cell suspensions from lungs of wild-type (WT) and ALCAM^−/−^ mice. FACS analysis using I/F8-Fc in combination with a fluorescently labeled secondary antibody detected ALCAM expression in LECs (CD45^−^CD31^+^podoplanin^+^) and blood vascular ECs (BECs: CD45^−^CD31^+^podoplanin^−^) of WT but not of ALCAM^−/−^ mice (Figures 1H–J), thereby proving the specificity of the antibody.

### ALCAM Blockade Reduces (Lymph)Angiogenic Processes *in vitro*

**Figure 2:**
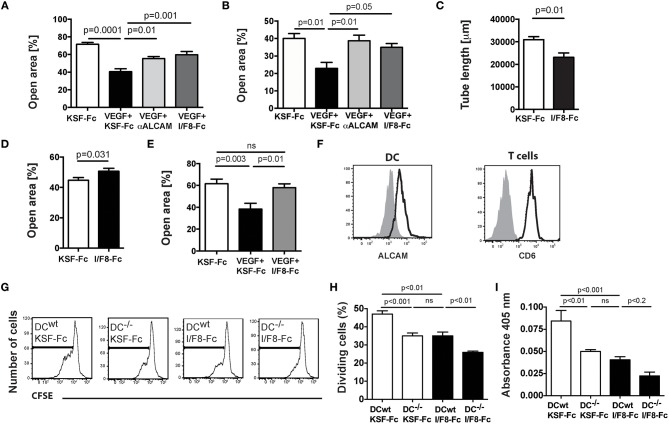
I/F8-Fc reduces (lymph)angiogenic processes and T cell activation *in vitro*. **(A–E)** Effects on *in vitro* (lymph)angiogenis. **(A,B)** A cell-free scratch was introduced into confluent monolayers of **(A)** human LECs or **(B)** HUVECs and the impact of I/F8-Fc or KSF-Fc control antibody on VEGF-A-induced scratch closure was analyzed after 24 and 12 h, respectively **(C)** Blocking ALCAM with I/F8-Fc reduced tube formation of human LECs. **(D,E)** A cell-free scratch was introduced into confluent monolayers of **(D)** murine MS-1 cell or **(E)** murine primary dermal LECs and the impact of I/F8-Fc on scratch closure was analyzed after 24 and 27 h, respectively. Data from 1 out of 3 to 4 similar experiments are shown in **(A–E)**. **(F–I)** Effects on *in vitro* T cell activation. WT or ALCAM^−/−^ BM-DCs were pulsed with OVA peptide in presence of LPS and co-incubated with CD4^+^ OTII cells in presence of I/F8-Fc or KSF-Fc control antibody. **(F)** FACS analysis demonstrating ALCAM and CD6 expression in BM-DCs and OTII cells, respectively. **(G,H)** Impact of I/F8-Fc treatment on T cell proliferation. **(G)** Representative FACS plots showing CFSE-dilution, as a readout of T cell proliferation. **(H)** Quantitation of proliferating cells. **(I)** T cell-mediated IFN-γ production was quantified in the cell culture supernatants. Data from 1 out of 4 similar experiments (*n* = 6 replicates) are shown in F-I. KSF-Fc: control antibody. I/F8-Fc: anti-ALCAM.

Next we tested the ability of I/F8-Fc to interfere with (lymph)angiogenic processes *in vitro*. Both a commercially available antibody blocking human ALCAM (12), as well as I/F8-Fc significantly reduced VEGF-A-induced migration of human LECs and of human umbilical vein ECs (HUVECs) in a scratch wound assay (Figures 2A,B). The ability of human LECs to form tube-like structures into collagen gels was significantly reduced in presence of I/F8-Fc (Figure 2C). I/F8-Fc also interfered with murine EC migration, as evidenced in scratch wound assays performed with blood vascular MS-1 cells and with primary murine dermal LECs (Figures 2D,E).

### ALCAM Blockade Reduces T Cell Activation *in vitro*

Recent *in vitro* studies revealed that ALCAM supports T cell activation by binding to the costimulatory molecule CD6 (9). In a competition ELISA I/F8-Fc significantly and dose-dependently reduced murine CD6-Fc binding to plate-bound murine ALCAM (Figures S3A,B). We next performed *in vitro* DC-T cell co-culture assays involving CD4^+^ T cells isolated from TCR-transgenic OTII mice (22) and WT or ALCAM^−/−^ bone marrow-derived DCs (BM-DCs) pulsed with peptide derived from ovalbumin (OVA) (Figures 2F–I). FACS analysis confirmed constitutive expression of CD6 in CD4^+^ OTII T cells and of ALCAM in BM-DCs (Figure 2F). Similarly to two recent reports (10, 11), we found that *in vitro* proliferation (Figures 2G,H) and IFN-γ production (Figure 2I) of OTII T cells were significantly reduced in co-cultures involving ALCAM^−/−^ DCs. Moreover, treatment of co-cultures involving WT DCs with I/F8-Fc reduced T cell proliferation and IFN-γ production to similar levels as observed in co-cultures of T cells and ALCAM^−/−^ DCs (Figures 2H,I). Thus, I/F8-Fc was capable of reducing murine T cell activation to comparable levels as seen in ALCAM deficiency.

### ALCAM Blockade Reduces Developmental Lymphangiogenesis

**Figure 3:**
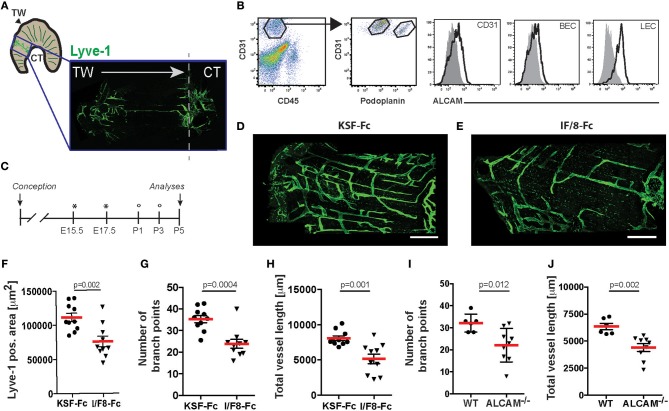
I/F8-Fc treatment reduces developmental lymphangiogenesis in the diaphragm to a similar degree as ALCAM deficiency. **(A)** Schematic representation of the diaphragm and of the area analyzed by LYVE-1 immunofluorescence in D-J. TW: thoracic wall; CT: central tendon. **(B)** ALCAM expression on diaphragmatic P5 BECs (CD45^−^CD31^+^podoplanin^−^) and LECs (CD45^−^CD31^+^podoplanin^+^) was confirmed by FACS. Data from 1 out of 2 similar experiments are shown. Specific stainings are shown as black lined and isotype control stainings as gray tinted histograms. **(C)** Schematic presentation of the antibody treatment protocol. Pregnant mice received I/F8-Fc or KSF-Fc antibody i.p. (300 μg) on day E15.5 and E17.5 (indicated by an asterisk). In addition, newborn mice received antibody i.p. (30 μg) on day P1 and P3 (indicated by a circle). **(D–H)** On P5 the diaphragm was collected and lymphatic vessels were visualized by LYVE-1 staining. **(D,E)** Representative stainings from diaphragms of KSF-Fc- or I/F8-Fc-treated animals. **(F–H)** Image-based morphometric analysis of **(F)** the LYVE-1^+^ area, **(G)** the number of branch points and **(H)** the total vessel length in the diaphragm of I/F8-Fc or KSF-Fc control-treated pups. Each dot represents the measurement made in one animal (*n* = 10). Data from 1 out of 3 similar experiments are shown in F-H. **(I,J)** Image-based morphometric analysis of **(I)** the number of branch points and **(J)** the total vessel length in the P5 diaphragm of WT and ALCAM^−/−^ mice. Data from 1 experiment (*n* = 6 or 9 mice) are shown in G-H. KSF-Fc: control antibody. I/F8-Fc: anti-ALCAM.

Given that ALCAM^−/−^ mice display a defect in the formation of the lymphatic network in various organs (12), we next investigated the ability of I/F8-Fc to block lymphangiogenesis *in vivo*. On the pleural side of diaphragmatic muscle segments, developing lymphatic vessels start to grow from E14.5 onwards from both the thorax wall and the central tendon. These vessels eventually form a mature lymphatic vasculature network, which can be analyzed after birth (23) (Figure 3A). FACS analysis performed on enzymatically-digested diaphragms of P5 old mice confirmed high ALCAM expression in LECs and to a lesser extent in BECs (Figure 3B). To investigate the impact of I/F8-Fc on lymphangiogenesis, pregnant WT mice were treated i.p. with I/F8-Fc or control KSF-Fc on embryonic day E15.5 and E17.5, and pups received additional antibody treatments on days P1 and P3 after birth (Figure 3C). Confocal analysis of the diaphragmatic muscle segments on P5 revealed a significant reduction in the LYVE-1^+^ lymphatic vessel area, the number of branch points, and in the total vessel length upon I/F8-Fc-treatment (Figures 3D–H). A strikingly similar defect in lymphatic network development was observed when analyzing the diaphragmatic muscle segments of P5 ALCAM^−/−^ mice (Figures 3I,J). Moreover, ALCAM-blockade also interfered with the development of the lymphatic network in the mesentery (Figure S4), resulting in similar changes as previously described for ALCAM deficiency (12). Overall, these data confirmed the ability of I/F8-Fc to specifically and potently interfere with developmental lymphangiogenesis.

### ALCAM Blockade Reduces DC Migration *in vitro*

**Figure 4:**
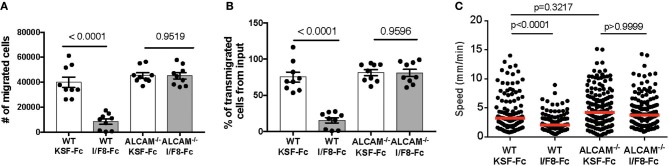
ALCAM blockade reduces DC transmigration and crawling on lymphatic endothelium *in vitro*. **(A,B)** imLECs were seeded on the lower surface of transwell inserts and grown to confluence. Transmigration of BM-derived WT or ALCAM^–/–^ BM-DCs across imLEC monolayers was investigated in presence of either KSF-Fc (control) or I/F8-Fc antibody. **(A)** Absolute numbers and **(B)** % of transmigrated DCs from the input. Each dot represents the value obtained from one transwell. Pooled data from 3 similar experiments (involving 3 transwells per condition per experiment) are shown in **(A,B)**. **(C)** DCs isolated from either WT or ALCAM^−/−^ mice were treated with I/F8-Fc or KSF-Fc antibody, followed by analysis of crawling speed on ALCAM-expressing imLEC monolayers. Each dot represents one DC. Pooled data from 2 to 3 similar experiments are shown. KSF-Fc: control antibody. I/F8-Fc: anti-ALCAM.

ALCAM was shown to mediate *in vitro* trans-endothelial migration of monocytes (7, 8) and T cells (6), but its role in DC migration through afferent lymphatics is unclear. To address whether ALCAM blockade would impact DC migration, we performed *in vitro* transwell assays involving ALCAM-expressing WT BM-DCs (Figure 2F) and ALCAM-expressing conditionally immortalized murine LECs (imLECs (24)—Figure 1G), which were grown to confluence on the lower surface of the transwell insert, to mimic basolateral-to-luminal transmigration. Treatment with I/F8-Fc significantly reduced transmigration of WT DCs (Figures 4A,B). Interestingly, the transmigration of ALCAM^−/−^ DCs was normal and could not be reduced by treatment with I/F8-Fc (Figures 4A,B). Similar findings were made when studying DC transmigration in the inverted setup, i.e., upon growing LECs on the upper surface of the transwell (Figure S5). *In vivo*, DCs that have transmigrated into lymphatic capillaries continue to actively crawl within the capillary lumen. They only detach to be passively transported with the lymph flow once they have reached contracting collecting vessel segments (25, 26). To investigate whether ALCAM might also contribute to intralymphatic crawling, we analyzed the impact of ALCAM blockade on DCs crawling on imLEC monolayers by time-lapse imaging. Treatment with I/F8-Fc effectively reduced the *in vitro* crawling speed of DCs on ALCAM-expressing imLEC monolayers (Figure 4C). Similarly to the results observed in DC transmigration (Figures 4A,B) ALCAM^−/−^ DCs migrated normally on imLECs, and their crawling speed was not affected by I/F8-Fc treatment (Figure 4C). Overall, these findings showed that I/F8-Fc treatment reduces transmigration and crawling of ALCAM-expressing WT DCs *in vitro*.

### ALCAM Blockade Delays Corneal Allograft Rejection

**Figure 5:**
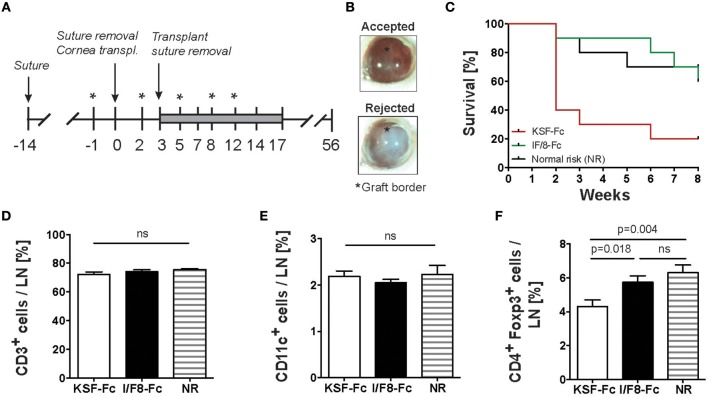
ALCAM blockade reduces corneal allograft rejection. **(A)** Schematic description of the corneal allograft rejection model. On day −14 three sutures were placed into the corneas of recipient BALB/c mice. On day 0, sutures were removed and the donor corneas from C57BL/6 mice were transplanted. Mice received I/F8-Fc or KSF-Fc control antibody i.p. (150 μg) on day −1, 2, 5, 8, and 12 (indicated by an asterisk). Additionally, mice were treated by topical application of antibody (3 μg) twice a day for 14 days (day 3–14; gray bar). Transplant survival was monitored over 8 weeks (day 0–56). Transplants were considered rejected when the cornea lost its transparency. **(B)** Representative images of a surviving and a rejected cornea. The graft border is indicated by an asterisk. **(C)** Impact of antibody treatment on corneal allograft survival. NR group: normal risk group, consisting of mice that received a cornea transplant without the previous induction of suture-induced inflammation. Statistical analysis: I/F8-Fc vs. KSF-Fc: *p* = 0.024; I/F8-Fc vs. NR: *p* = 0.77; KSF-Fc vs. NR: *p* = 0.019. **(D–F)** FACS analysis was performed on eye-draining cervical LNs of all mice at the end of the study. Percentage of **(D)** CD45^+^CD3^+^ T cells, **(E)** CD45^+^ CD11c^+^ DCs **(F)** and CD45^+^CD4^+^Foxp3^+^ Tregs in cervical LNs. Pooled data from 2 experiments with a total of 10 mice /group are shown.

Since our data demonstrated an impact of ALCAM blockade on (lymph)angiogenesis, T cell activation and DC migration, we reasoned that therapeutic targeting of ALCAM might be an effective approach for preventing corneal allograft rejection. To investigate our hypothesis, we performed experiments in an established corneal allograft rejection model in mice (27). In this model, a high-risk condition for allograft rejection is generated by placing sutures into the corneas of recipient BALB/c mice. This induces inflammation and the formation of blood and lymphatic vessels into the usually avascular cornea. Two weeks after suture placement, corneas of C57BL/6 mice are transplanted into recipient BALB/C mice (Figure 5A). FACS analysis performed on single cell suspensions of suture-inflamed murine corneas revealed that ALCAM was expressed on corneal LECs but not BECs (Figure S6). To investigate the impact of ALCAM-blockade on graft survival, treatment with I/F8-Fc or with KSF-Fc control was started 1 day prior to cornea transplantation and continued for 2 weeks. The rejection process was monitored over a total of 8 weeks post-transplantation (Figures 5A,B). In this setup, the survival of the transplanted allografts was significantly increased in the group treated with ALCAM-blocking antibody IF/8-Fc as compared to the control-treated group (Figure 5C). Notably, the graft survival rate was comparable to the one observed in the normal-risk control group, which had received allograft transplantation into avascular corneas. FACS analysis of the cornea-draining cervical LNs 8 weeks after cornea transplantation did not reveal any difference in the relative percentages of T cells and DCs, but a significant increase in the percentage of regulatory T cells (T_regs_) in the I/F8-Fc-treated group, likely reflecting the less inflamed, more tolerance-inducing conditions (Figures 5D–F).

### ALCAM Blockade Does Not Reduce Corneal (Lymph)Angiogenesis but Results in the Accumulation of Corneal DCs

**Figure 6:**
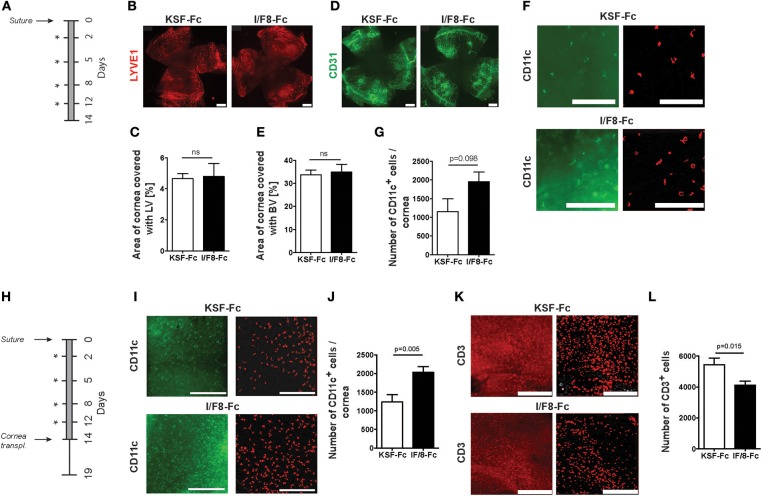
ALCAM blockade results in accumulation of corneal DCs. **(A)** Schematic description of the suture model. Three sutures were placed into the corneas. Mice received I/F8-Fc or KSF-Fc control antibody i.p. (150 μg) on day 2, 5, 8, and 12 (indicated by an asterisk) as well as antibody-containing eye drops (3 μg) twice a day for 14 days (gray bar). Corneas were analyzed by whole-mount immunofluorescence on day 14 (*n* = 5/group). **(B–G)** Representative immunofluorescence images and quantifications of the percentage of corneal area covered with **(B,C)** LYVE-1^+^ lymphatic vessels (LVs), **(D,E)** CD31^+^ blood vessels (BVs), scale bars: 500 μm, and **(F,G)** CD11c^+^ DCs, scale bars: 100 μm. Images on the left in **(F)** show CD11c^+^ cells (green) detected by immunofluorescence. Images on right show corresponding cells (red) identified by gray-image threshold analysis, which was used for quantification **(G)**. **(H)** Schematic representation of the suture/allograft model: three sutures were placed into the corneas and animals were treated as in **(A)**. On day 14 corneal transplantation was performed. Five days after transplantation (day 19), corneas were analyzed by whole-mount immunofluorescence (*n* = 10/group). **(I–L)** Representative images and quantifications of the percentage of corneal area covered with **(I,J)** CD11c^+^ DCs and **(K,L)** CD3^+^ T cells. DC and T cell quantification in **(I–L)** was performed as in **(F,G)**. Images on the left in **(I,K)** depict DC and T cells as seen in immunofluorescence; images on the right (in red) as identified by gray-image threshold analysis. Scale bars: 100 μm.

To better understand how ALCAM blockade contributed to cornea survival, we investigated the impact of I/F8-Fc treatment on (lymph)angiogenesis and on leukocyte infiltration in the corneal suture model. For this, sutures were placed into the corneas and the animals were treated for 2 weeks with either I/F8-Fc or KSF/Fc (Figure 6A). Surprisingly, whole-mount immunofluorescence revealed no difference in the lymphatic vessel coverage of the cornea, indicating that anti-ALCAM treatment did not substantially impact inflammatory lymphangiogenesis (Figures 6B,C). Also no difference in angiogenesis was detected (Figures 6D,E). By contrast, a near-significant increase in corneal CD11c^+^ cells was observed in I/F8-Fc treated mice, suggesting a role of ALCAM blockade in the retention of antigen-presenting DCs in the cornea (Figures 6F,G). To further investigate into this hypothesis, we repeated the suture experiment, treating (recipient) mice with I/F8-Fc or KSF-Fc for 2 weeks, but this time transplanting corneal allografts into the pre-vascularized corneal beds at the end of the 2 weeks' treatment period, to further exacerbate the inflammatory stimulation (Figure 6H). When analyzing the entire corneas on day 5 after transplantation, a marked and significant increase in DC numbers present in corneas of I/F8-Fc-treated as compared to KSF-Fc-treated mice was observed (Figures 6I,J). By contrast, T cell numbers were significantly reduced (Figures 6K,L). Overall, these data suggest that ALCAM-blockade supported allograft survival in mice by reducing the emigration of antigen-presenting DCs from corneas via lymphatic vessels to dLNs.

### ALCAM Blockade Reduces DC Emigration From Human Skin Biopsies

**Figure 7:**
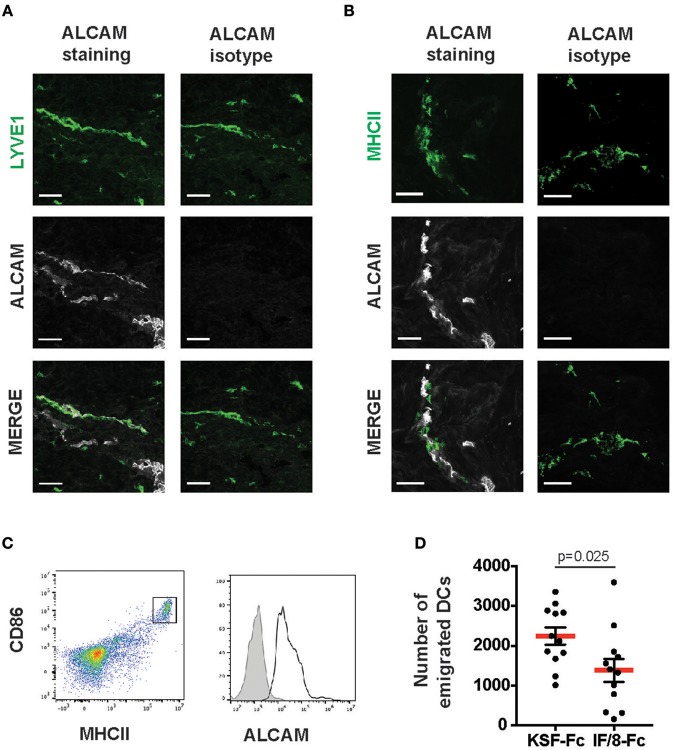
ALCAM blockade reduces DC emigration from human skin. Human skin sections were stained for **(A)** LYVE-1 and **(B)** MHCII, in combination with either an ALCAM-specific or the corresponding isotype control antibody. ALCAM reactivity was observed in **(A)** all LYVE-1^+^ vessels and **(B)** in some MHCII^+^ cells. Scale bars: 50 μm. **(C)** Human skin punch biopsies were incubated in culture medium and the presence of ALCAM on emigrated CD86^+^MHCII^hi^ cells was analyzed after 24 h. Data from 1 representative of a total of 3 experiments are shown. **(D)** Human skin DC emigration experiment. Punch biopsies of human skin were incubated in culture medium in presence of either I/F8-Fc or of KSF-Fc control antibody, and cells emigrated into the culture medium were analyzed by FACS 24 h later. In **(C)** representative FACS plots show the gating scheme. Migratory DCs were identified as CD86^+^MHCII^hi^ cells and were quantified with the help of counting beads. Each dot represents one biopsy (*n* = 10 per condition).

To gain further evidence for this hypothesis, we investigated the impact of ALCAM blockade on DC emigration from human skin biopsies. Previous studies have shown that upon *in vitro* culture of murine and human skin biopsies, DCs mainly exit from the biopsy tissue into the culture medium by migrating into and through lymphatic vessels (28–30). As previously reported, ALCAM is not expressed in the vasculature of murine skin (12, 14), but it is highly expressed in the human dermal lymphatic vasculature (Figure 7A). Moreover, we detected ALCAM expression in MHCII^+^ cells in human skin (Figure 7B). Following 24 h of *in vitro* culture of human dermal punch biopsies, the culture medium contained a population of emigrated MHCII^+^CD86^+^DCs, which all expressed ALCAM (Figure 7C). The number of emigrated DCs was significantly reduced when skin biopsies were cultured in medium containing I/F8-Fc but not KSF-Fc (Figure 7D). Therefore, ALCAM blockade with I/F8-Fc was able to reduce DC emigration from human skin.

### Endothelial Cells and Antigen-Presenting Cells in Inflamed Human Cornea Express ALCAM

**Figure 8:**
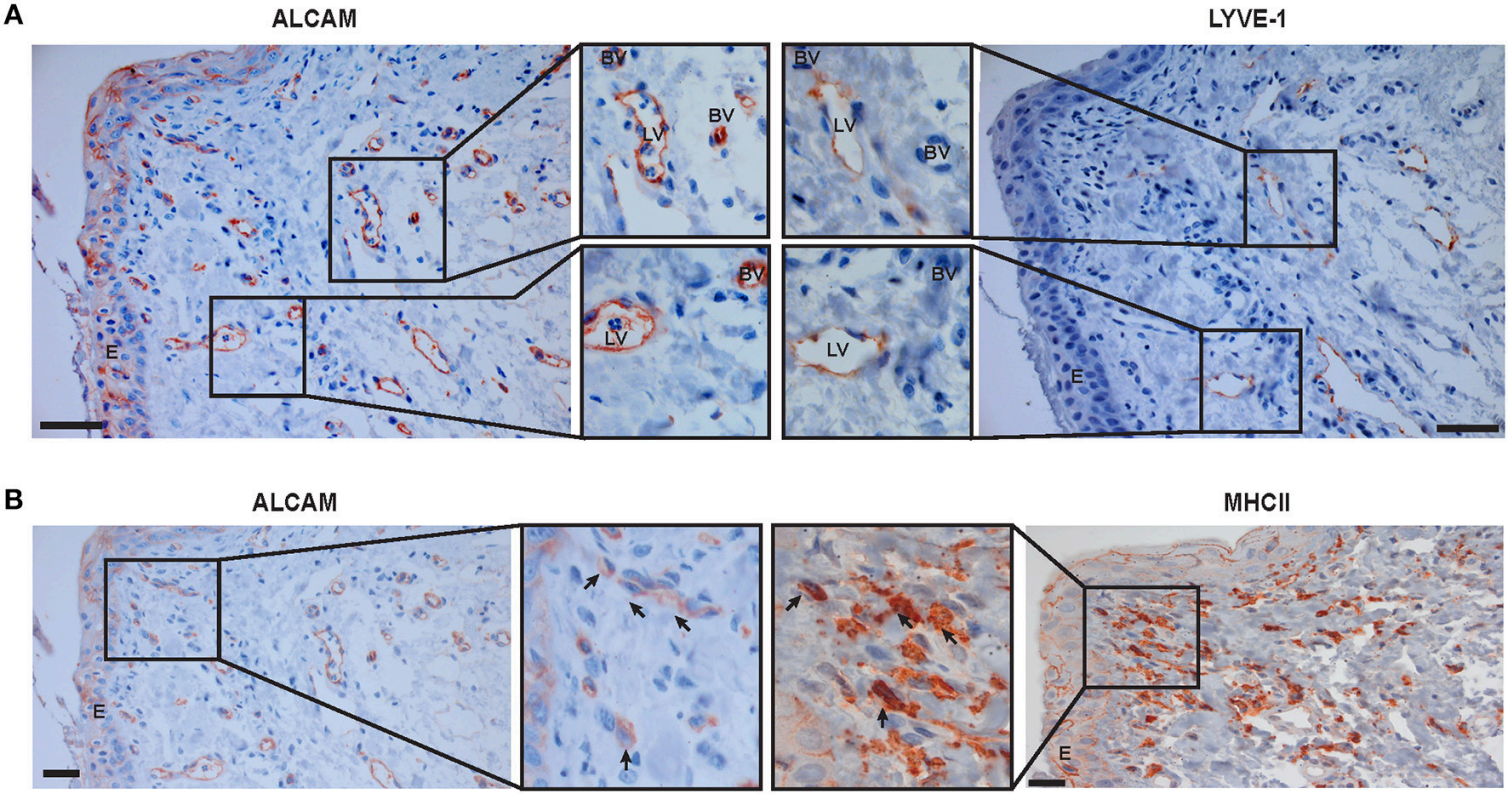
ALCAM is expressed in pathologic blood and lymphatic vessels in neovascularized human corneas and in corneal DCs. Serial sections of inflamed human corneas obtained from high risk transplant patients were stained for **(A)** ALCAM and LYVE-1 and **(B)** ALCAM and MHC-II. Magnifications of the boxed areas are shown in the center part of the Figure. LV, Lymphatic vessel; BV, Blood vessel, E, epithelium. Arrows indicate specific staining. Scale bar: 20 μm. Representative images from 3 stained neovascularized corneas are shown.

To investigate the translational potential of ALCAM blockade in human corneal allograft rejection, we analyzed ALCAM expression in the neo-vasculature of rejected human corneas derived from patients prior to cornea transplantation. In these samples, we detected ALCAM expression in newly-formed corneal blood and lymphatic vessels (Figure 8A). Moreover, co-staining for MHCII confirmed expression of ALCAM in antigen-presenting cells of human corneas (Figure 8B).

## Discussion

In this study, we characterized and validated a monoclonal antibody with blocking activity toward murine ALCAM and used this tool to identify ALCAM as a novel mediator of DC migration and as a promising therapeutic target for preventing corneal allograft rejection. Additionally, our *in vitro* findings further showed that treatment with I/F8-Fc significantly reduced lymphangiogenesis and T cell activation, suggesting that the I/F8-Fc-induced delay of corneal allograft rejection might have been caused by its impact on all of the above-mentioned processes.

Surprisingly, although I/F8-Fc treatment reduced developmental lymphangiogenesis to the levels seen in ALCAM^−/−^ mice, we did not observe any reduction in inflammation-induced corneal lymphangiogenesis upon I/F8-Fc treatment. Given that our FACS-based analysis confirmed ALCAM expression in corneal LECs albeit not in BECs, the inability of I/F8-Fc to reduce corneal lymphangiogenesis is likely not explained by the absence of ALCAM expression on corneal lymphatics. On the other hand, it is possible, that under inflammatory conditions, the contribution of ALCAM to lymphangiogenesis might be less strong as compared to its role during developmental lymphangiogenesis. For example, in inflammation, the role of ALCAM might be compensated by up-regulation of other EC-expressed adhesion molecules or mediators. Alternatively, the massively trapped DCs and macrophages could also have acted as a powerful source of pro-lymphangiogenic growth factors, thereby compensating for the inhibitory effect of ALCAM blockade (31).

The fact that we detected increased DC numbers in the corneas of I/F8-Fc-treated mice, both in the suture model as well as in transplanted corneal allografts indicates that the observed therapeutic effect of ALCAM blockade was at least in part due to a decrease in DC emigration from the cornea via lymphatic vessels. In support of this hypothesis our results further showed that ALCAM blockade significantly reduced *in vitro* transmigration and crawling of DCs on LEC monolayers. The fact that ALCAM^−/−^ DC transmigrated and crawled normally and were not impacted by ALCAM-blockade suggests that in absence of ALCAM DCs might upregulate compensatory adhesion or signaling molecules to support migration. For example, it was recently reported that genetic deletion of ALCAM induces upregulation of RAGE (receptor for advanced glycation end products) (32), a molecule involved in inflammatory signaling and also in leukocyte trafficking. However, considering that ALCAM is highly expressed by human and murine DCs (9, 33, 34) and also by LECs of all human and most murine tissues analyzed so far (with the exception of murine skin), we assume that most *in vivo* DC migration processes are ALCAM-dependent. In support of this assumption we also found that treatment with I/F8-Fc significantly reduced emigration of DCs from human skin explants into the culture medium. Taken together, these data strengthen our hypothesis that the accumulation of DCs in corneas of I/F8-Fc-treated mice was caused by inhibition of DC emigration via lymphatics to dLNs.

We also observed that ALCAM blockade significantly reduced T cell numbers in corneas. Given that ALCAM has been implicated in T cell transmigration across blood vascular endothelium (6), it is possible that the observed reduction was caused by a direct impact on T cell extravasation into the inflamed cornea. However, similarly to another study (8), we did not observe any effect of ALCAM blockade in reducing *in vitro* transmigration of T cells across LEC and BEC monolayers (data not shown). Alternatively, the reduced T cell numbers detected in I/F8-Fc-treated corneas could have been indirectly caused by reduced DC migration and, consequently, reduced priming of alloreactive T cells in cornea-draining LNs. Last but not least, in our *in vitro* experiments ALCAM blockade also reduced T cell activation by antigen-presenting DCs, likely by interfering with ALCAM-CD6 interactions. Given that ALCAM^−/−^ mice display reduced T cell activation and T cell mediated inflammatory responses in experimental mouse models of antigen-induced food allergy or asthma (10, 11), it is possible that the I/F8-mediated delay in corneal allograft rejection can in part be attributed to direct effects on T cell activation.

In support of the translational relevance of our findings we detected ALCAM expression in MHCII^+^ DCs present in inflamed human corneas as well as in pathologic corneal blood and lymphatic vessels. The latter is in agreement with two recent studies, which identified ALCAM in ECs isolated from human corneas (35, 36). Up to now, the main treatment options for prevention of corneal allograft rejection are corticosteroids. However, these are often not sufficient to achieve complete inhibition and may cause unwanted side effects such as cataracts or elevated intraocular pressure (15). While anti-(lymph)angiogenic therapies targeting the VEGF-A or VEGF-C signaling pathways (17, 37, 38) or the insulin receptor substrate-1 (39, 40) have been brought forward in many (pre)clinical studies as promising therapies in corneal allograft rejection, only few other targets have been described so far (19, 41, 42). Interestingly, another study recently demonstrated that blockade of CCL21/CCR7 signaling, i.e., the best described signaling axis supporting DC migration (43), promoted graft survival in a low-risk corneal transplantation setup (42). Surprisingly, our findings show that even under high-risk conditions, i.e., in presence of a fully vascularized recipient bed, the post-operative blockade of ALCAM improved graft survival to a normal risk level and thereby overcame the risk of graft rejection associated with the presence of blood and lymphatic vessels. This suggests ALCAM blockade to act via a “functional lymphatic blockade,” i.e., having no direct effect on lymphangiogenesis, but functionally preventing DCs from using lymphatic vessels to reach the dLN and induce an immune response.

In conclusion, our findings identify ALCAM as a novel therapeutic target for preventing corneal allograft rejection in high-risk patient populations. Moreover, given the documented contribution of ALCAM to (lymph)angiogenesis, DC migration, and T cell activation, ALCAM blockade might represent a therapeutic strategy for treating other immune-mediated inflammatory conditions, such as solid organ transplant rejection, allergy or autoimmune diseases.

## Experimental Procedures

### Cloning, Expression, and Purification of I/F8-Fc and KSF-Fc

For cloning of I/F8-Fc and KSF-Fc the respective scFv sequence was amplified from previously cloned scFv I/F8 with specificity for human ALCAM (20) and scFv KSF with specificity for hen egg lysozyme (21), and linked to a murine Fc fragment (C_H_2 and C_H_3 domains of murine IgG_1_) amplified from a commercial cDNA (Source BioScience, Berlin, Germany). The following primer sets were used appending a part of the 13 amino acid long hinge region (*GTGCCCAGGGATTGTGGTTGTAAGC*CTTGCATATGTACA) at the N-terminus: scFv I/F8: 5′ TCCTCCTGTTCCTCGTCGCTGTGGCTACAGGTgtgcacTCGGAGGTGCAGCTGTTGGAGTTGGG ′3 and 5′ GGGACCAAGCTGACCGTCCTAGGC*GTGCCCAGGGATTGTGGTTGTAA* ′3; KSF:5′TCCTCCTGTTCCTCGTCGCTGTGGCTACAGGTgtgcacTCGGAGGTGCAGCTGTTGGAGTCTGGG 3′ and 5′ GGGACCAAGCTGACCGTCCTAGGC*GTGCCC AGGGATTGTGGTTGT AA* ′3. At the same time the murine Fc fragment was amplified using the following primers: 5′ GTGCCCAGGGATTGTGGTTGTAAGCCTTGCATATGTACAGTCCCAGAAG ′3 and 5′ TTTTCCTTTTGCGGCCGCTCATTAAGCTATTTACCAGGAGAGTGGGAGAGG ′3 introducing a stop codon. Each scFv sequence was PCR-assembled with the sequence of the murine Fc using the primer sets containing the HindIII and NotI restriction sites: I/F8-Fc: 5′ CCCAAGCTTGTCGACCATGGGCTGGAGCCTGATCCTCCTGTTCCTCGTCGCTGTGGC ′3 and 5′ CCTCTCCCACTCTCCTGGTAAATAGCTTAATGAGCGGCCGCAAAAGGAAAA ‘3 and KSF-Fc: 5‘CCCAAGCTTGTCGACCATGGGCTGGAGCCTGATCCTCCTGTTCCTCGTCGCTGT-GGC′3 and 5′ CCTCTCCCACTCTCCTGGTAAATAGCTTAATGAGCGGCCGCAAAAGGAAAA ′3. The amplification product was double-digested with HindIII/NotI (both from NEB, Ipswich, MA, USA) and ligated into the pcDNA3.1 (+) vector (Life Technologies, Carlsbad, CA, USA). A monoclonal cell line was generated as previously described (44). Briefly, CHO–S cells (44) were transfected with the plasmids and clones expressing high levels of antibody were screened by ELISA coated with either ALCAM-His (45) or hen egg lysozyme (Sigma-Aldrich, Buchs, St. Louis, Missouri, USA), followed by detection with anti-murine IgG coupled to horse radish peroxidase (Thermo Scientific, Waltham, MA, USA). Large-scale production was performed by culturing best-producing clones in PowerCHO-2CD (Lonza), followed by purification over Protein A columns (Sino Biological, Beijing, China), as described previously (46). Purified antibodies were dialyzed into PBS, passed through 0.22 μm PES filters (TPP, Trasadingen, Switzerland) and stored at −80°C after snap-freezing with liquid nitrogen. Purified antibodies were analyzed by SDS-PAGE and liquid chromatography on a Superdex 200 10/300 GL column (GE Healthcare, Chalfont St Giles, UK).

### BIAcore

BIAcore analysis was performed on a BiaCore3000 (GE Healthcare, Chalfont St Giles, UK) using high density coated CM_5_ sensor chips (GE Healthcare) coated with human CD166(28-501)-His (45) or murine CD166 (1-527)-His (Sino Biological, Beijing, China) as described (47).

### Competition ELISA

96-well plates (Nunc, Roskilde, Denmark) were coated overnight at 4°C with purified murine CD166(1-527)-His (5 μg/ml, Sino Biological) blocked with PBS containing 2% milk powder and subsequently incubated for 2 h at RT with a fixed amount of murine CD6-Fc (1 μg/ml; R&D Systems, Minneapolis, MN, USA) and decreasing concentrations of I/F8-Fc or KSF-Fc in PBS (4 to 0.25 μg/ml). Bound CD6-Fc was detected using horse radish peroxidase-coupled anti-human IgG antibody (Life Technology). The ELISA was developed by adding 3,3′,5,5′-tetramethylbenzidine liquid substrate (BioLegend, San Diego, California, USA), followed by absorbance measurements at 450 and 570 nm.

### Mice

For *in vivo* experiments related to the cornea, 6–8 week old female BALB/c mice and C57BL/6 mice were purchased from Charles River Laboratories (Sulzfeld, Germany). For all other experiments, C57BL/6-J mice were purchased from Janvier (Genest-Saint-Isle, France), whereas ALCAM^−/−^ mice (48) and T cell receptor (TCR) transgenic OTII mice (22) were bread in the ETH Rodent Center HCI.

### DC-T Cell Co-culture Assay

CD4^+^ T cells were isolated from OTII mice (22) as previously described (49). In brief, LN and spleen single-cell suspensions were incubated with anti-CD4^+^-coupled magnetic beads (Miltenyi Biotech, Gladbach, Germany), followed by positive separation on an LS MACS column and separator (all from Miltenyi Biotech). Before co-culturing isolated CD4^+^ T cells were stained with 2 μM CFSE (Invitrogen) in PBS for 12 min at 37°C. Bone marrow-derived DCs (BM-DCs) were generated as described previously (49) by culturing in DC medium containing RPMI 1,640, 10% FBS, 15 nM HEPES, 1 mM sodium pyruvate, penicillin (100 U/ml), streptomycin (100 μg/ml), L-glutamin (2 nM) (all purchased from GIBCO, Zug, Switzerland) 50 μg β-mercaptoethanol (Sigma) and GM-CSF derived from the supernatant of myeloma cells (X63 Ag8.653) transfected with murine GM-CSF cDNA (50). 1 × 10^4^ DCs and 5 × 10^4^ CFSE-labeled OTII CD4^+^ T cells were co-cultured in the presence of 100 nM OVA peptide (residues 323–339 of ovalbumin, GenScript, Piscataway, NJ, USA) and 10 ng/ml LPS (Axxora, San Diego, CA, USA) and I/F8-Fc or KSF-Fc (10 μg/ml), respectively in 200 μl of the following medium: RPMI-1640 medium (Life Technologies) containing 10 % FBS (Life Technologies), 50 μM β-mercaptoethanol (Sigma-Aldrich), 15 mM HEPES (Life Technologies), 1 mM Na-pyruvate (Life Technologies), 1% antibiotic-antimycotic solution (Life Technologies), and 1% L-glutamine (Life Technologies). After 3 days, cells were stained with anti-CD4-APC (Biolegend) and CFSE dilution was analyzed by FACS. For quantification of IFN-**γ** production, half of the cells were transferred to a new well and fresh media was added. On day 4, a 96-well plate (Nunc, Roskilde, Denmark) was coated overnight at 4°C with 50 μl of 5 μg/ml anti-CD3 antibody (Biolegend). The next day, cell suspensions of co-cultures were transferred onto anti-CD3 coated wells. After 48 h, the supernatant was harvested and IFN-γ quantified by ELISA (see next chapter).

### IFN-γ ELISA

96-well plates (Nunc) were coated overnight at 4°C with 5 μg/ml anti-IFN-**γ** capture antibody (BioLegend—clone R4-6A2) and blocked with 2.5% FCS (Life Technologies) in PBS. Subsequently, the cell supernatant and a known IFN-γ (PeproTech) concentration were added. After 4 h the plate was washed with PBS containing 0.005% Tween-20 followed by incubation with a biotinylated anti-IFN-γ detection antibody (Biolegend—clone XMG1.2) for 1 h. Wells were washed and subsequently incubated for 1 h with streptavidin-coupled alkaline phosphatase (GE Healthcare). For detection the substrate solution SIGMAFAST p-Nitrophenyl phosphate (Sigma-Aldrich) was added. After stopping the reaction with 3 M NaOH the absorbance was measured at 405 nm.

### Endothelial Cell Culture and Assays

All ECs were cultured on plates coated with 10 μg/ml of collagen type I (Advanced BioMatrix, San Diego, CA, USA) and 10 μg/ml fibronectin (Merck Millipore, Darmstadt, Germany). Human dermal LECs (Lonza, Walkersville, MD, USA) and HUVECs (Promocell, Heidelberg, Germany) were cultured in EGM-2MV medium without VEGF-A (Lonza). Mouse Mile Sven-1 (MS-1) cells (51) and conditionally immortalized LECs (imLECs) (24) were cultured in imLEC medium containing 40% F12-Ham, 40% DMEM (low glucose), 20% FBS (all from Gibco), L-glutamin (2 nM; Fluka), 10 μg/ml EC mitogen (AbD Serotec, Duesseldorf, Germany), 56 μg/ml heparin (Sigma), and antibiotic antimycotic solution (1x; Fluka, Buchs, Switzerland). Primary mouse dermal LECs (Cell Biologics, Chicago, IL, USA) were cultured in the same medium supplemented with 1 μg/ml hydrocortisone (Sigma-Aldrich).

### Transmigration Assay

Inserts from 24-well transwell plates (Costar, Sigma-Aldrich) were coated with collagen type 1 (Advanced Biomatrix) and fibronectin (Merck Millipore) (10 μg/ml each) and seeded with 175'000 imLECs in imLEC medium on the lower side or on the upper side of the transwell membrane, in analogy to the setup originally described by Johnson et al. (52). The medium was exchanged after 6 h and again the next day. Two days later, 1 × 10^5^ LPS-activated BM-DCs (WT or ALCAM^−/−^) were added to the top well and left to migrate toward the bottom well containing 100 ng/ml of CCL21 (PeproTech) in DMEM/F12 medium containing 2% FCS. After 4 h, transmigrated DCs were stained with anti-mouse CD11c-APC (BioLegend) and quantified by FACS. For assessing antibody efficacy, WT DCs and LECs were both pre-incubated with I/F8-Fc or KSF-Fc (10 μg/ml) for 30 min at 37°C prior to performing the experiment in presence of antibodies.

### Cell Crawling Assay

Channeled chamber slides (μ-Slide VI^0.4^, IBIDI, Martinsried, Germany) were coated with collagen type 1 (Advanced Biomatrix) and fibronectin (Merck Millipore) (10 μg/ml each) and seeded with 30,000 imLECs in imLEC medium. Cells were cultured for a further 2 days with exchange of the imLEC medium 1–2 h after seeding and again the next day. On the day of the experiment, WT or ALCAM^−/−^ LPS-matured BM-DCs (always >90% CD11c^+^) were labeled with 0.5 μM CellTracker™ Deep Red Dye (DR; Thermo Fisher Scientific; Waltham, MA, USA) in RPMI 1640 for 15 min, washed three times with PBS, and incubated in DC medium for 30–40 min. DCs were subsequently washed with PBS and centrifuged over an FBS gradient to remove any dead cells. Before each experiment, the purity and maturation state of LPS-matured BM-DCs was confirmed by flow cytometry, staining with anti-CD11c-APC, anti-I-A/I-E-BV421, anti-CD80-FITC and anti-CD86-PE antibodies (all Biolegend). Analysis was performed on a Cytoflex S (Beckman Coulter) with CytExpert software, and analyzed with FlowJo software 10.4.0. (Treestar). DR-labeled LPS-activated BM-DCs and confluent imLEC monolayers were incubated with I/F8-Fc or KSF-Fc (10 μg/ml) for 30 min. Thirty thousand pre-blocked BM-DCs were directly added to pre-blocked imLEC monolayers and after 20 min, chambers were rinsed twice with imLEC medium to remove non-adherent cells. After a further 10–20 min equilibration in imLEC medium at 37°C, cells were imaged in imLEC medium using time lapse imaging on a fluorescent microscope (Nikon Eclipse Ti-E, Tokyo, Japan) equipped with a Hamamatsu ORCA-Flash4.0 CCD camera (Hamamatsu, Japan) and a 20x objective (NA: 0.75, Nikon). Phase contrast and Cy5 fluorescence images were captured every 30 s for 50–60 min. Movies were analyzed using IMARIS software (v7.1.1, Bitplane, Zurich, Switzerland). Cell tracks were generated by an automatic algorithm and verified manually. Cells that clustered or migrated for <10 min were excluded from analysis. Cell speed was calculated as total track length/track duration.

### Tube Formation Assay

Confluent human LEC monolayers grown in 24-well plates were cultured for 24 h in starvation medium [EBM-2 (Lonza) containing 2% FCS (Life Technologies) and 1% antibiotic-antimycotic solution (Life Technologies)]. Subsequently, wells were overlaid with 0.5 ml of neutralized isotonic bovine dermal collagen type I (1 mg/ml; Advanced BioMatrix) containing 10 μg/ml of either I/F8-Fc or KSF-Fc antibody. Representative images (3 per well) of the tubes were taken after 16–18 h using an Axiovert 200 M microscope and a 5 × objective (NA of 0.12) (Carl Zeiss, Inc.). The images were acquired with the Axiovision software (version 4.7.1, Carl Zeiss) and the total length of all tube-like structures in the pictures of each well was measured using a semi-manual ImageJ-based script developed in-house. The person performing the image-based analysis was blinded to allocation of the different treatment groups.

### Scratch-Wound Assay

Confluent monolayers of human LECs or murine MS-1 cells grown in 24-well plates were cultured for 24 h in starvation medium. In the case of human LECs and HUVECs, starvation medium consisted of EBM-2 (Lonza) containing 2% FCS (Life Technologies) and 1% antibiotic-antimycotic solution (Life Technologies). In the case of primary dermal LECs and MS-1, starvation medium consisted of DMEM medium (Life Technologies) supplemented with 5% FCS (Life Technologies) and 1% antibiotic-antimycotic solution (Life Technologies). Subsequently, two cross-shaped scratches were made in each well. Monolayers were washed with PBS and 500 μl of starvation medium supplemented with αALCAM (R&D Systems), I/F8-Fc, isotype control, or KSF-Fc (all 10 μg/ml) in combination with VEGF-A (20 ng/ml; PeproTech) was added. Pictures were taken immediately after scratching and 12–24 h later using an Axiovert 200 M microscope and a 5 × objective (NA of 0.12) (Carl Zeiss, Inc.). Afterwards, the percentage of the surface area closed after 16–24 h was calculated using TScratch software (53).

### FACS Analysis

For FACS analysis *in vitro* cultured cells and tissues were prepared as previously described (12). In the case of the lung, tissue was digested in collagenase IV-containing Grey's Balanced Salt Solution (GBSS, Sigma-Aldrich). Cell suspensions were stained with anti-mouse CD31-APC, anti-mouse CD45-PerCP and podoplanin-PE (all from Biolegend) to identify LECs (CD45^−^CD31^+^podo^+^) and BECs (CD45^−^CD31^+^podo^−^), respectively. ALCAM expression was detected by staining with goat anti-mouse ALCAM antibody (R&D Systems) and corresponding AlexaFluor488- or PE-labeled secondary antibodies (Invitrogen). Some FACS experiments were performed using I/F8-Fc (2 μg/ml) or KSF-Fc (2 μg/ml) followed by incubation with anti-mouseAlexaFluor488 or -PE-labeled secondary antibodies (Invitrogen, Carlsbad, CA, USA). Data were acquired on a BD FACSCanto (BD Bioscience, Franklin Lake, NJ, USA) using FACSDiva software (BD Bioscience) and analyzed with FlowJo software 8.7.1. (Treestar, Ashland, TN, USA).

### Blockade of Developmental (Lymph)Angiogenesis

I/F8-Fc or of KSF-Fc (300 μg) were administered i.p. to pregnant C57BL/6-J females on embryonic day (E) 15.5 and E17.5, followed by i.p. treatment (30 μg) of pups postnatally on day (P)1 and P3. Pups were sacrificed on P5.

### Whole-Mount Immunofluorescence and Morphometric Analysis of Diaphragm and Mesentery

Harvested tissues were treated as previously described (12). The following primary antibodies were used: rat anti-mouse CD31 (BD Bioscience), goat anti-mouse Prox-1 (R&D Systems), rabbit anti-mouse LYVE-1 (AngioBio, San Diego, CA, USA), goat anti-mouse LYVE-1 (R&D Systems), or anti-smooth muscle actin coupled to Cy3 (Sigma-Aldrich). The next day whole mounts were incubated for 2 h with AlexaFluor488, 594, or 647-conjugated secondary antibodies (all from Invitrogen). Whole-mount z-stacks were acquired either on a Leica TCS SP8 (Leica Microsystems, Wetzlar, Germany) or on a SP2 AOBS confocal microscope (Leica). Images were acquired using the Leica Application Suite LASX software (version 1.8.0.13370; Leica Microsystems) or the Leica confocal software (version 2.61; Leica Microsystems), respectively. Lymphatic vessels on the pleural side of the diaphragmatic muscle were analyzed as described previously (23). Per diaphragm two segments of the diaphragmatic muscle were analyzed with ImageJ (NIH, Bethesda, MD, USA) in order to quantify the LYVE-1 positive area, the average branch length and the number of branches, as well as the total vessel length. The number of branch points was quantified manually. All image-based quantifications were performed in a blinded manner.

### Corneal Suture Model

The corneal suture model was performed as previously described (19, 27). In brief, female BALB/c mice were anesthetized with ketamine (120 mg/kg) and xyalzine (20 mg/kg) and three interrupted intrastromal sutures (10–0 nylon, Sharepoint Surgical Specialities Corp., Wyomissing, PA) were placed in the corneal stroma and left in place for 14 days. During this time animals were treated with eye drops containing either I/F8-Fc or KSF/Fc (3 μg/drop) twice a day and additionally received intraperitoneal antibody injections (150 μg) on day 2, 5, 8, and 12 after suture placement. Mice were sacrificed and corneas were histologically analyzed on day 14.

### Corneal Transplantation Model

#### Allograft Survival Study

Female recipient BALB/c mice received 3 intrastromal figure eight sutures, as described above, to induce neovascularization. After 13 days mice were intraperitoneally injected with either I/F8-Fc or KSF-Fc (150 μg). The next day mice were subjected to corneal transplantation as described previously (27). In brief, mice were anesthetized with ketamine (120 mg/kg) and xyalzine (20 mg/kg). Donor corneas from C57BL/6 mice were excised (1.5 mm), placed in the graft bed and secured with eight interrupted sutures (10-0 nylon, Sharepoint Surgical Specialities Corp., Wyomissing, PA, USA). After transplantation antibiotic treatment with FLOXAL (Bausch&Lomb) was administered locally and eyelids were sutured shut (8-0 nylon, Sharepoint Surgical Specialities Corp.) for 3 days. Animals were treated with either I/F8-Fc or KSF/Fc administered i.p. on day 2, 5, 8, and 12 post-transplantation (150 μg/injection), and additionally with eye drops that were administered twice a day (3 μg/dose), starting day 3 post-transplantation, once the eyelid sutures had been removed. Surgical sutures were removed on day 7 post-transplantation, and grafts were graded for opacity twice a week until the end of the experiment on day 56 after transplantation. Investigators were blinded to the treatment group allocation during the entire experiment. Clinical scores were assigned, as described previously (27): 0, clear; 1, minimal, superficial (non-stromal) opacity; pupil margin, and iris vessels readily visible through the cornea; 2, minimal, deep (stromal) opacity; pupil margins and iris vessels visible; 3, moderate stromal opacity; only pupil margin visible; 4, intense stromal opacity; only a portion of pupil margin visible; and 5, maximum stromal opacity; anterior chamber not visible. Grafts with opacity scores of 2 or greater after 2 weeks were considered as rejected.

#### Analysis of Leukocyte Infiltration Study

Female recipient BALB/c mice received 3 intrastromal figure eight sutures, as described above. Animals were treated with eye drops containing either I/F8-Fc or KSF/Fc twice a day (3 μg/drop) and additionally received i.p. antibody injections (150 μg) on day 2, 5, 8, and 12 after suture placement. On day 14 corneal transplantations were performed as described above. Animals were sacrificed 5 days post-transplantation and corneas were harvested for histological analysis.

### Histological Analysis of Mouse Cornea

Corneas were harvested at the endpoint of the experiment. Corneas were fixed in acetone, rinsed in PBS and blocked with 2% bovine serum albumin. Vascular staining was performed with FITC-conjugated CD31 (Santa Cruz Biotechnology), FITC-conjugated CD11c (Biolegend), unconjugated rabbit anti mouse CD3 (Abcam) or LYVE-1 (AngioBio co.) overnight. Subsequently, corneas were washed in PBS, stained with anti-rabbit Cy3 (Jackson Immuno Research), washed and mounted onto slides using fluorescence mounting media (DAKO).

Quantification analysis was performed using multiple digital images that were automatically assembled of the flatmounts acquired by a Olympus BX53 microscope (Olympus, Tokyo, Japan) and Cell∧f Software (Olympus), as previously described (54). In brief, CD31 positive structures were defined as blood vessels, while LYVE-1 positive structures were defined as lymphatic vessels. Vessel coverage was quantified and normalized to total cornea area to obtain percentage coverage. Quantification of immune cell infiltration was also performed by gray image threshold analysis using Cell∧f software (Olympus), as described (54, 55). In brief, the total area with positive CD11c or CD3 signals in the cornea was divided by the average size of a single positive cell, yielding the overall cell number in the region of interest. The average cell size was determined from manual measurements of the areas of 100 single cells.

### FACS-Based Detection of ALCAM Expression by Murine Corneal Endothelial Cells

Female BALB/c mice underwent suture-induced neovascularization as described above, but received no additional treatment. For each experiment (*n* = 2) vascularized corneas from 10 animals were harvested and pooled on day 14 after suture placement. Corneas were cut into small pieces, digested with Liberase TL (Roche, Switzerland; 2.5 mg/ml in PBS, incubated at 37°C for 30 min) and passed through a cell strainer. Single cell suspensions were stained with rat anti-mouse CD31-APC, rat anti-mouse CD45-PE/Cy7, and Syrian hamster anti-mouse podoplanin-PE (all from Biolegend), to identify LECs (CD45^−^CD31^+^podoplanin^+^) and BECs (CD45^−^CD31^+^podoplanin^−^). ALCAM was detected by staining with I/F8-Fc (2 μg/ml) or KSF-Fc (2 μg/ml), followed by incubation with anti-mouse-AlexaFluor488 secondary antibody (Invitrogen). Samples were acquired on a BD FACSCanto (BD Bioscience, USA), using FACSDiva software (BD Bioscience) and analyzed with FlowJo software (Treestar, Ashland, TN, USA).

### Human Skin Emigration Experiments

Human skin punch biopsies (Ø 3–5mm) were placed in RPMI 1,640 medium (Gibco) supplemented with 2 mM glutamine (Gibco), 10% heat inactivated fetal calf serum and 100 μg/mL Normocin (InvivoGen). From each donor two (or four) biopsies with identical diameter and thickness were cultured in medium, supplemented with either 10 μg/ml of I/F8-Fc or KSF/Fc (same number of biopsies per condition, to ensure equal average punch sizes for both conditions). Crawled out cells in the culture medium were quantified 24 h later by FACS. For this, biopsies were removed and flow-count fluorospheres (Beckman Coulter) as well as anti-HLA-DR (mouse anti-human HLA-DR FITC; Miltenyi Biotec) and anti-CD86 (mouse anti-human CD86-APC; Miltenyi Biotec) antibodies were added to the medium. In separate experiments, the presence of ALCAM was additionally determined on CD86^+^HLA-DR^+^ DCs that had emigrated from untreated biopsies by co-staining with polyclonal goat anti-ALCAM (R&D Systems, AF1172) or corresponding isotype control (R&D Systems), followed by AlexaFluor594-conjugated secondary antibodies (Invitrogen). Samples were acquired on a Cytoflex S apparatus (Beckman Coulter) using CytExpert software and analyzed with FlowJo software 10.4.0. (Treestar).

### Statistical Data Analysis

Statistical analysis was performed using Prism 6 (GraphPad Software, La Jolla, CA, USA). Normally distributed data was analyzed using the Student's *t-*test and are presented as mean ± standard error (SEM). For comparisons of the means of more groups one-way ANOVA was used. Cell tracking data are represented as medians and were analyzed using a Kruskal-Wallis test for multiple comparisons. Kaplan-Meyer curves were analyzed using Log-rank test. Differences were considered statistically significant when *p* < 0.05. In the case of the human skin emigration assay (Figure 6D) two significant outliers (1 per treatment condition) were identified based on Grubbs' test and excluded.

## Ethics Statement

Mouse studies related to the cornea were approved by State Agency for Nature, Environment and Consumer Protection North Rhine-Westphalia (LANUV NRW, license number 84-02.04.2016A055) and conformed to the Association for Research in Vision and Ophthalmology's Statement for the Use of Animals in Ophthalmology and Vision Research. All other mouse studies were approved by the Cantonal Veterinary Office Zurich (license numbers ZH110/12, ZH088/15 & ZH268/2014). The immunohistochemical stainings of human corneas were performed on specimens from clinical routine histology and were approved by the Institutional Review Board of the University of Cologne (Reference Number 14–246). Human skin emigration experiments were performed with tissue samples obtained from the University of Zurich Biobank, funded by the University of Zurich University Research Priority Program (URPP) in translational biology. The study was approved by the Institutional Review Board of the University of Zurich (KEK-ZH-Nr. 2015–0209). All patients (skin donors) signed informed consent in accordance with the Biobank project (EK No. 647).

## Author Contributions

A-HW designed research, performed research, analyzed data, and wrote the paper. A-CS, DI, AG-O, and MH designed research, performed research, and analyzed data. MV, PS, AR, ES, and VS performed research and analyzed data. DN, CC, MF, and SF provided essential reagents and discussed data. EG designed research and analyzed and discussed data. FB and CH designed research, analyzed data, and wrote the paper.

### Conflict of Interest Statement

The authors declare that the research was conducted in the absence of any commercial or financial relationships that could be construed as a potential conflict of interest.
