# Supplementary material for: ALCAM Mediates DC Migration Through Afferent Lymphatics and Promotes Allospecific Immune Reactions
Source: Front Immunol. 2019 Apr 12;10:759. doi: 10.3389/fimmu.2019.00759 (PMC6473055; doi:10.3389/fimmu.2019.00759)
Supplement: Supplementary file 1 [file Data_Sheet_1.PDF]

Supplemental Figure and Figure Legends

Figure S1: Characterization of I/F8-Fc and MAB656 binding to murine and human ALCAM. (**A**) human LECs (hLECs), (**B**) conditionally immortalized murine LECs (imLECs) or murine bone marrow-derived, LPS-matured DCs from (**C**) WT or (D) ALCAM$^{-/-}$ mice were stained for ALCAM expression using either I/F8-Fc (i.e. the antibody described in this study) or the commercial mouse-anti-human ALCAM monoclonal antibody MAB656 (R&D Systems) and the corresponding isotype control antibodies. Specific stainings are shown as black lined and isotype control stainings as grey tinted histograms. MAB656 consistently recognized human ALCAM on hLECs but - in contrast to I/F8-Fc - failed to bind to murine ALCAM on either imLECs or WT DCs. Representative stainings from > 4 experiments are shown (performed with 2 different batches of MAB656).

Figure S2: Nucleotide sequence of I/F8-Fc and KSF-Fc antibody (**A**) Schematic representation of the pcDNA3.1 plasmid coding for the scFv-Fc antibodies. (**B/C**) Nucleotide sequence of I/F8-Fc (B) and KSF-Fc (**C**) containing the secretion peptide, the two variable domains that are connected via a 14 amino acid (aa) long linker and the two constant domains joined to the variable domains via a 13 aa long hinge region. The antibody sequence is flanked by the restriction sites HindIII and NotI, which were used for cloning.

Figure S3: I/F8-Fc blocks ALCAM interaction with CD6. (**A**) Schematic representation of the possible ALCAM interactions occurring during the formation of the immunological synapse. (**B**) I/F8-Fc effectively competes with murine CD6-Fc for binding to recombinant murine ALCAM. Wells that had been coated with recombinant ALCAM were incubated with recombinant CD6-Fc (1 μg/ml) in the presence of increasing concentrations of I/F8-Fc (0.125 – 4 μg/ml). Bound CD6-Fc was detected by ELISA. Pooled data from 4 similar experiments are shown.

Figure S4: I/F8-Fc reduces developmental lymphangiogenesis in the mesentery. Pregnant mice received I/F8-Fc or KSF-Fc antibody i.p. (300 μg) on day E15.5 and E17.5. Once born, pups received antibody i.p. (30 μg) on day P1 and P3. On P5 the mesenteries were collected and lymphatic vessels were visualized by Prox-1 staining. (**A**) Representative stainings of the Prox-1^{+} mesenteric vasculature of KSF-Fc- or I/F8-Fc-treated animals. Scale bars: 100 μm. (**B-D**) Image-based morphometric analysis of (**B**) the average branch length, (**C**) the number of branch points and (**D**) the number of lymphatic ring structures in the mesentery of I/F8-Fc- or KSF-Fc control-treated pups. Each dot represents the measurement made in one animal (n=6-7).

Figure S5: ALCAM blockade reduces DC transmigration through lymphatic endothelium *in vitro.* imLECs were seeded on the upper surface of transwell inserts and grown to confluence. Transmigration of BM-derived WT or ALCAM$^{-/-}$ BM-DCs across imLEC monolayers was investigated in presence of either KSF-Fc (control) or I/F8-Fc antibody. (**A**) Absolute numbers and (**B**) % of transmigrated DCs from the input. Each dot represents the value obtained from one transwell. Pooled data from 3 similar experiments (involving 3 transwells per condition per experiment) are shown.

Figure S6: FACS-based analysis of ALCAM expression in the mouse corneal neovasculature. Neovascularization was induced in murine corneas by placing sutures. 14 days later, corneas from 10 mice were pooled, enzymatically digested and single-cell suspensions stained for FACS-based analysis of CD45, CD31 and podoplanin expression. (**A**) General gating strategy applied. BECs and LECs were identified by gating on CD45$^-$CD31$^+$podoplanin$^-$ (BECs) or CD45$^-$CD31$^+$podoplanin$^+$ (LECs) cells. (**B**) ALCAM expression in BECs and LECs.

Supplemental Figures

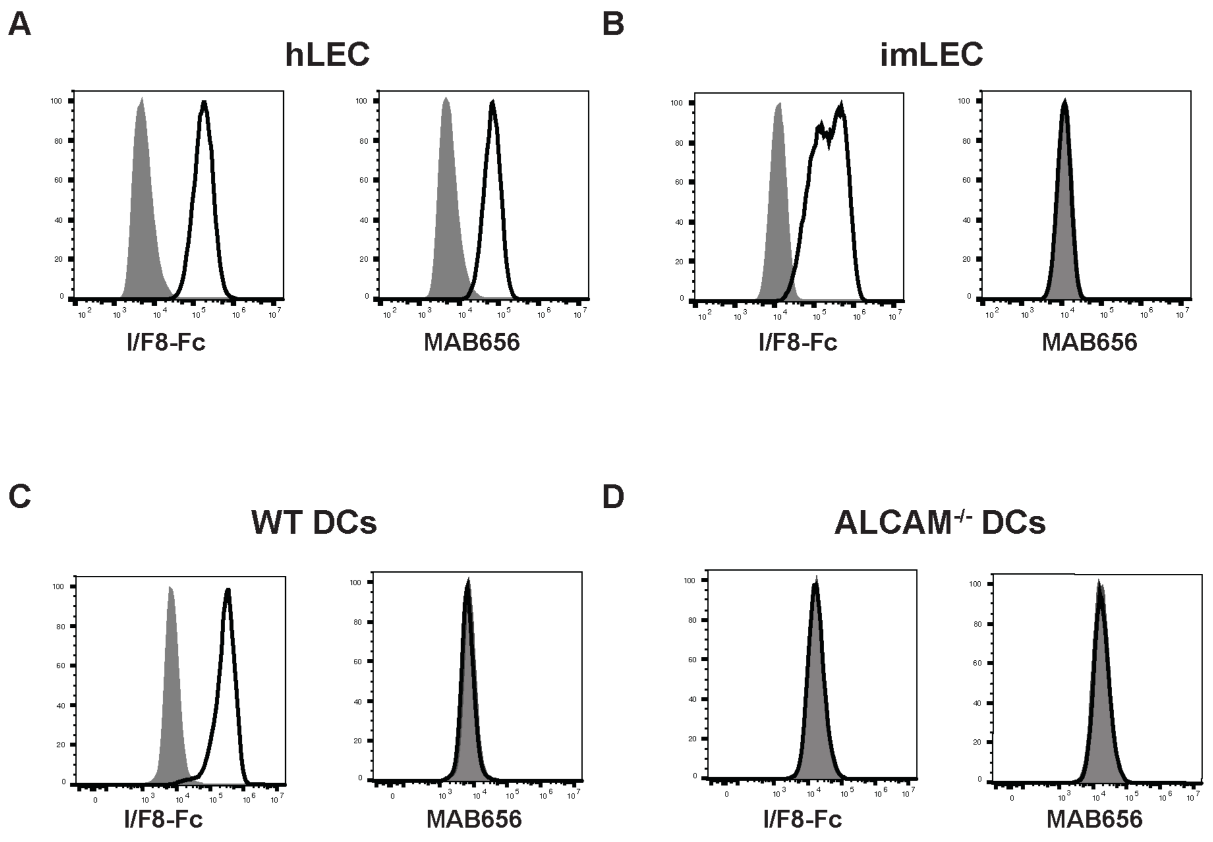

Figure S1

A

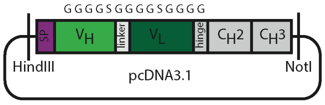

B

CCCAAGCTTGTCGACCATGGGCTGGAGCCTGATCCTCCTGTTCCTCGTCGCTGTGGCTACAG
GTGTGCACTCGGAGGTGCAGCTGGTGGAGTCTGGGGGAGGCTTGGTACAGCCTGGGGGGTCC
CTGAGACTCTCCTGTGCAGCCTCTGGATTCACCTTTAGCAGCTATGCCATGAGCTGGGTCCG
CCAGGCTCCAGGGAAGGGGCTGGAGTGGGTCTCAGCTATTAGTGGTAGTGGTGGTAGCACAT
ACTACGCAGACTCCGTGAAGGGCCGGTTCACCATCTCCAGAGACAATTCCAAGAACACGCTG
TATCTGCAAATGAACAGCCTGAGAGCCGAGGACACGGCCGTATATTACTGTGCGAAAGGGTA
TGTGGCTTTTGACTCCTGGGGCCAGGGAACCCTGGTCACCGTGTCGAGAGGTGGAGGCGGTT
CAGGCGGAGGTGGCTCTGGCGGTGGCGGATCGTCTGAGCTGACTCAGGACCCTGCTGTGTCT
GTGGCCTTGGGACAGACAGTCAGGATCACATGCCAAGGAGACAGCCTCAGAAGCTATTATGC
AAGCTGGTACCAGCAGAAGCCAGGACAGGCCCCTGTACTTGTCATCTATGGTAAAAACAACC
GGCCCTCAGGGATCCCAGACCGATTCTCTGGCTCCAGCTCAGGAAACACAGCTTCCTTGACC
ATCACTGGGGCTCAGGCGGAAGATGAGGCTGACTATTACTGTAACTCCTCTCCCCCTTTTAG
TGCGGAGGTGGTATTCGGCGGAGGGACCAAGCTAACCGTCCTAGGC*GTGCCCAGGGATTGTG*
*GTTGTAAGC*CTTGCATATGTACAGTCCCAGAAGTATCATCTGTCTTCATCTTCCCCCCAAAG
CCCAAGGATGTGCTCACCATTACTCTGACTCCTAAGGTCACGTGTGTTGTGGTAGACATCAG
CAAGGATGATCCCGAGGTCCAGTTCAGCTGGTTTGTAGATGATGTGGAGGTGCACACAGCTC
AGACAAAACCCCGGGAGGAGCAGTTCAACAGCACTTTCCGTTCAGTCAGTGAACTTCCCATC
ATGCACCAGGACTGGCTCAATGGCAAGGAGTTCAAATGCAGGGTCAACAGTGCAGCTTTCCC
TGCCCCCATCGAGAAAACCATCTCCAAAACCAAAGGCAGACCGAAGGCTCCACAGGTGTACA
CCATTCCACCTCCCAAGGAGCAGATGGCCAAGGATAAAGTCAGTCTGACCTGCATGATAACA
GACTTCTTCCCTGAAGACATTACTGTGGAGTGGCAGTGGAATGGGCAGCCAGCGGAGAACTA
CAAGAACACTCAGCCCATCATGGACACAGATGGCTCTTACTTCGTCTACAGCAAGCTCAATG
TGCAGAAGAGCAACTGGGAGGCAGGAAATACTTTCACCTGCTCTGTGTTACATGAGGGCCTG
CACAACCACCATACTGAGAAGAGCCTCTCCCACTCTCCTGGTAAA**TAG**CTTAATGAGCGGCC
GCAAAAGGAAAA

C

CCCAAGCTTGTCGACCATGGGCTGGAGCCTGATCCTCCTGTTCCTCGTCGCTGTGGCTACAG
GTGTGCACTCGGAGGTGCAGCTGTTGGAGTCTGGGGGAGGCTTGGTACAGCCTGGGGGGTCC
CTGAGACTCTCCTGTGCAGCCTCTGGATTCACCTTTAGCAGCTATGCCATGAGCTGGGTCCG
CCAGGCTCCAGGGAAGGGGCTGGAGTGGGTCTCAGCTATTAGTGGTAGTGGTGGTAGCACAT
ACTACGCAGACTCCGTGAAGGGCCGGTTCACCATCTCCAGAGACAATTCCAAGAACACGCTA
TATCTGCAAATGAACAGCCTGAGAGCCGAAGACACGGCCGTATATTACTGTGCGAAATCGCC
TAAGGTGTCGCTTTTTGACTACTGGGGCCAGGGAACCCTGGTCACCGTCTCGAGTGGTGGAG
GCGGTTCAGGCGGAGGTGGCTCTGGCGGTGGCGGATCGTCTGAGCTGACTCAGGACCCTGCT
GTGTCTGTGGCCTTGGGACAGACAGTCAGGATCACATGCCAAGGAGACAGTCTCAGAAGCTA
TTATGCAAGCTGGTACCAGCAGAAGCCAGGACAGGCCCCTGTACTTGTCATCTATGGTAAAA
ACAACCGGCCCTCAGGGATCCCAGACCGATTCTCTGGCTCCAGCTCAGGAAACACAGCTTCC
TTGACCATCACTGGGGCTCAGGCGGAAGATGAGGCTGACTATTACTGTAACTCCTCTCCCCT
GAATCGGCTGGCTGTGGTATTCGGCGGAGGGACCAAGCTGACCGTCCTAGGC*GTGCCCAGGG*
*ATTGTGGTTGTAAGC*CTTGCATATGTACAGTCCCAGAAGTATCATCTGTCTTCATCTTCCCC
CCAAAGCCCAAGGATGTGCTCACCATTACTCTGACTCCTAAGGTCACGTGTGTTGTGGTAGA
CATCAGCAAGGATGATCCCGAGGTCCAGTTCAGCTGGTTTGTAGATGATGTGGAGGTGCACA
CAGCTCAGACAAAACCCCGGGAGGAGCAGTTCAACAGCACTTTCCGTTCAGTCAGTGAACTT
CCCATCATGCACCAGGACTGGCTCAATGGCAAGGAGTTCAAATGCAGGGTCAACAGTGCAGC
TTTCCCTGCCCCCATCGAGAAAACCATCTCCAAAACCAAAGGCAGACCGAAGGCTCCACAGG
TGTACACCATTCCACCTCCCAAGGAGCAGATGGCCAAGGATAAAGTCAGTCTGACCTGCATG
ATAACAGACTTCTTCCCTGAAGACATTACTGTGGAGTGGCAGTGGAATGGGCAGCCAGCGGA
GAACTACAAGAACACTCAGCCCATCATGGACACAGATGGCTCTTACTTCGTCTACAGCAAGC
TCAATGTGCAGAAGAGCAACTGGGAGGCAGGAAATACTTTCACCTGCTCTGTGTTACATGAG
GGCCTGCACAACCACCATACTGAGAAGAGCCTCTCCCACTCTCCTGGTAAA**TAG**CTTAATGA
GCGGCCGCAAAAGGAAAA

Figure S2

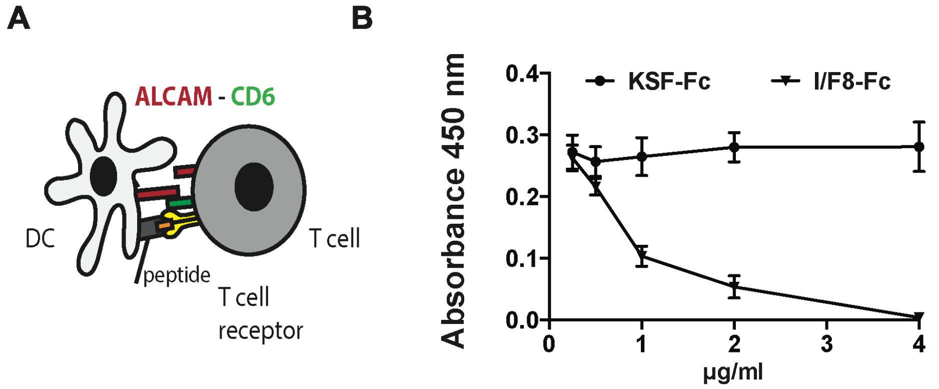
A
B
ALCAM - CD6
DC
T cell
peptide
T cell
receptor
Absorbance 450 nm
KSF-Fc
I/F8-Fc
0.4
0.3
0.2
0.1
0.0
0
1
2
3
4
µg/ml

Figure S3

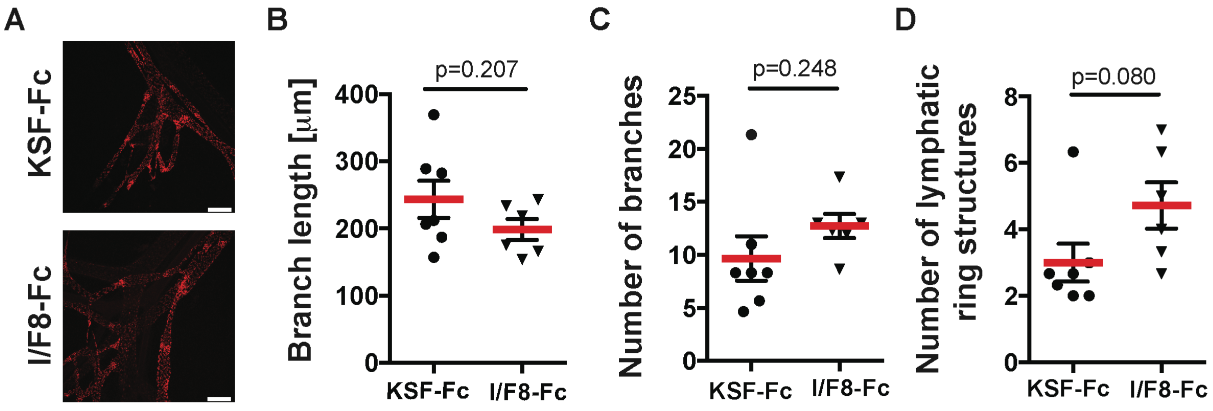
A
KSF-Fc
I/F8-Fc
B
p=0.207
Branch length [μm]
400
300
200
100
0
KSF-Fc
I/F8-Fc
C
p=0.248
Number of branches
25
20
15
10
5
0
KSF-Fc
I/F8-Fc
D
p=0.080
Number of lymphatic ring structures
8
6
4
2
0
KSF-Fc
I/F8-Fc

Figure S4

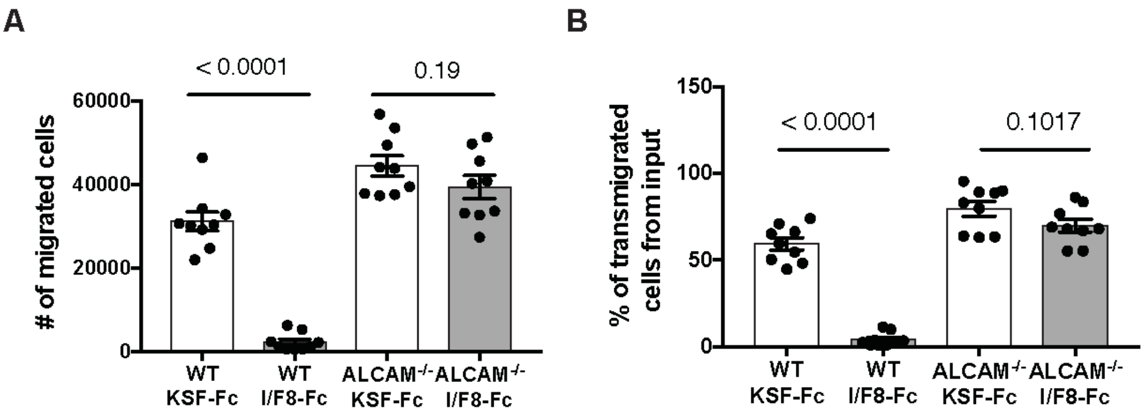
A
B
< 0.0001
0.19
# of migrated cells
60000
40000
20000
0
WT KSF-Fc
WT I/F8-Fc
ALCAM-/- KSF-Fc
ALCAM-/- I/F8-Fc
< 0.0001
0.1017
% of transmigrated cells from input
150
100
50
0

Figure S5

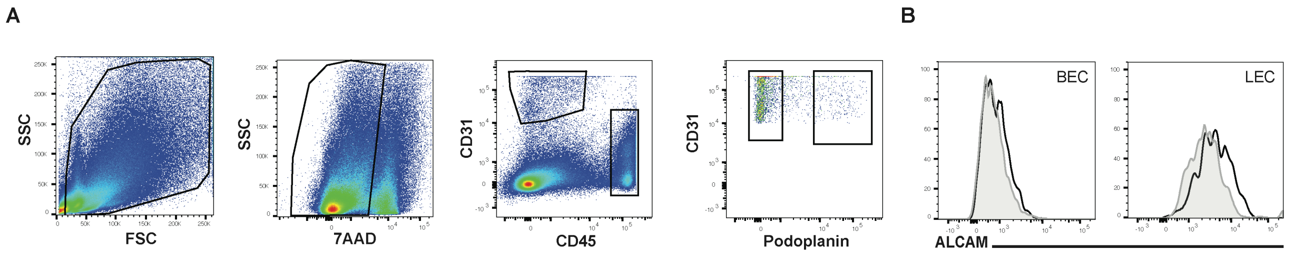

Figure S6
